# The rs1803274 polymorphism of the *BCHE* gene is associated with an increased risk of coronary in-stent restenosis

**DOI:** 10.1186/s12872-015-0128-8

**Published:** 2015-10-24

**Authors:** L. Pleva, P. Kovarova, L. Faldynova, P. Plevova, S. Hilscherova, J. Zapletalova, P. Kusnierova, P. Kukla

**Affiliations:** Department of Clinical Studies, Medical Faculty, University of Ostrava, Syllabova 19, Ostrava, Zabreh 703 00 Czech Republic; Department of Cardiovascular Diseases, University Hospital Ostrava, Ostrava, Czech Republic; Department of Biomedical Sciencies, Medical Faculty, University of Ostrava, Ostrava, Czech Republic; Blood Center, University Hospital of Ostrava, Ostrava, Czech Republic; Department of Medical Genetics, University Hospital Ostrava, Ostrava, Czech Republic; Department of Medical Biophysics, Palacky University, Olomouc, Czech Republic; Department of Laboratory Medicine, University Hospital Ostrava, Ostrava, Czech Republic

## Abstract

**Background:**

We sought to identify gene polymorphisms that confer susceptibility to in-stent restenosis after coronary artery bare-metal stenting in a Central European population.

**Methods:**

160 controls without post–percutaneous coronary intervention in-stent restenosis were matched for age, sex, vessel diameter, and diabetes to 160 consecutive cases involving in-stent restenosis of the target lesion within 12 months. Using real time polymerase chain reaction and melting-curve analysis, we detected 13 single-nucleotide polymorphisms in 11 candidate genes - rs1803274 (*BCHE* gene)*,* rs529038 (*ROS1*), rs1050450 (*GPX1*), rs1800849 (*UCP3*), rs17216473 (*ALOX5AP*), rs7412, rs429358 (*ApoE*)*,* rs2228570 (*VDR*), rs7041, rs4588 (*GC*)*,* rs1799986 (*LRP1*) and rs2228671 (*LDLR*)*.* Multivariable logistic regression was used to test for associations.

**Results:**

The rs1803274 polymorphism of *BCHE* was significantly associated with in-stent restenosis (OR 1.934; 95 % CI: 1.181–3.166; *p* = 0.009). No association was found with the other studied SNPs.

**Conclusions:**

The A allele of rs1803274 represents a risk factor for in-stent restenosis in Central European patients after percutaneous coronary intervention with bare-metal stent implantation.

## Background

Coronary stent implantation has significantly improved percutaneous coronary intervention (PCI). It has enabled management of early complications of plane balloon angioplasty and prevention of elastic recoil and constrictive remodeling, decreasing the frequency of restenosis after PCI. However, with these improvements has come a new complication: in-stent restenosis (ISR) arising from neointimal hyperplasia. The clinical incidence of ISR after bare-metal stent (BMS) implantation is about 20–35 % and currently represents one of the main limitations of coronary angioplasty [1]. The use of drug-eluting stents has led to a further decrease of ISR occurrence to 5–10 % [1]. The known risk factors for ISR include diabetes mellitus, renal insufficiency, lesion length (20 mm), small vessel diameter (3 mm), treatment of complex lesions (B2/C) [2], chronic total occlusions, venous bypasses, ostial or bifurcation lesions, stent undersizing or underexpansion and necessary implantation of more stents [1]. The increasing number of patients undergoing coronary interventions has led to attempts to find further biochemical and genetic risk factors that would enable more targeted treatment.

Based on a literature review, for the current study, single nucleotide polymorphisms (SNPs) of some candidate genes were selected for having been associated with ISR in different populations (Japanese and North American) or in patients with repeated restenosis, including *BCHE* - butyrylcholinesterase gene, *ROS1* - the closest homolog of the v-Ros oncogene of the avian sarcoma, *GPX1* - glutathione-peroxidase-1 gene, *UCP3* - uncoupling protein 3 gene, and *ALOX5AP* - arachidonate 5-lipoxygenase-activating protein gene. Our aim was to assess associations in our Central European population [3–5]. Furthermore, we focused on a group of genes affecting metabolic processes, including genes of apolipoprotein E (*APOE*), vitamin D receptor (*VDR*), vitamin D binding protein (*GC*), and two genes of the low density lipoprotein receptor family, low density lipoprotein receptor related protein 1 (*LRP1*) and low density lipoprotein receptor (*LDLR*) which may be involved not only in atherosclerosis but also in inflammation and may influence proliferation and migration of vascular smooth muscle cells (VSMCs), key players in the development of ISR [6–11].

## Patient groups and methods

We included 160 consecutive patients treated at the cathlab of the University Hospital Ostrava, Czech Republic, between the years 2010 and 2013, with ISR arising within 12 month after implantation of a bare-metal stent. The control group was searched in the Czech National PCI Registry and consisted of 160 matched patients treated at our cathlab with identical main demographic and clinical risk factors (sex, age, diabetes mellitus, implanted stent diameter ±0.5 mm) in whom ISR was excluded using multi-slice (MS-CT) coronarography scheduled at 12 months after the implantation.

In order to determine frequency of the studied SNPs in the general Czech population, genotyping was also performed in 200 persons (100 Czech man-woman pairs aged 26–66 years).

### Selective coronarography

Selective coronarography was performed in a Siemens Axiom device (Forchheim, Germany) using the standard method from the right or left radial approach with 5 F diagnostic catheters and the contrast medium Iomeron 400. Quantitative coronary angiography was performed, and percent diameter stenosis (% DS) was calculated. ISR was defined as a diameter stenosis ≥50 % in the stented segment.

### Multi-slice (MS) CT coronarography

All patients were examined without the need for previous premedication with beta-blockers in the Siemens Somatom Definition AS+ device (Forchheim, Germany), a single-source CT scanner in a 128-slice configuration. The maximum intensity projection (MIP) reconstructions and automatic software Vessel analysis were used for evaluation of the lumen. Homogeneous enhancement (visually similar to the CT attenuation in the reference vessels) inside the stent lumen was considered to be normal or unrelated to ISR.

### Genotyping of polymorphisms

The evaluated SNPs are listed in Table 1. The polymorphisms are described according to the HGVS nomenclature [12], and their description corresponds to the latest version available in the NCBI and Ensembl gene databases.

**Table 1:** Selected single nucleotide polymorphisms

Polymorphism			
Gene	Coding DNA level	Protein level	NCBI database
*BCHE*	c.1699G>A	p.Ala567Thr	rs1803274
*ROS1*	c.6637G>A	p.Asp2213Asn	rs529038
*UCP3*	c.-238C>T		rs1800849
*GPX1*	c.599C>T	p.Pro200Leu	rs1050450
*ALOX5AP*	c.117–5723G>A		rs17216473
APOE*2	c.526C>T	p.Arg176Cys	rs7412
APOE*4	c.388 T>C	p.Cys130Arg	rs429358
*GC*	c.1296 T>G	p.Asp432Glu	rs7041
*GC*	c.1307C>A	p.Thr436Lys	rs4588
*VDR*	c.2 T>C	p.Met1Thr	rs2228570
*LRP1*	c.300C>T	p.Asp100=	rs1799986
*LDLR*	c.81C>T	p.Cys27=	rs2228671

Genomic DNA was extracted using the MagNA Pure Compact Instrument (Roche, Switzerland). The real-time PCR assays for genotyping of rs1803274 (*BCHE*), rs529038 (*ROS1*), rs1050450 (*GPX1*), and rs1800849 (*UCP3*) were performed on the Rotor-Gene 3000A instrument (Corbett Research/Qiagen, Netherlands) with dual-labeled hydrolysis TaqMan® probes (Eastport, Czech Republic; TIB MolBiol, Germany) and followed by allelic discrimination and/or scatter analysis.

Primers and hybridization probes (TIB MOLBIOL, Germany) were designed to detect rs17216473 (*ALOX5AP*), rs2228570 (*VDR*), rs7041, rs4588 (*GC*), rs2228671 (*LDLR*), and rs1799986 (*LRP1*) genotypes using melting curve analysis on the LightCycler 480 II system (Roche, Switzerland). Primer and probe sequences are available on request.

Using these two methods, sample genotype was determined comparing the curves to positive samples whose genotype was verified by Sanger sequencing using the ABI Big Dye Terminator Cycle Sequencing Detection Kit v.3.1 (Applied Biosystems, USA) and genetic analyzer ABI3130 (Applied Biosystems, USA). Sanger sequencing was also used to determine the genotype of samples with inconclusive result of real-time PCR assay or melting curve analysis.

The ApoE*2, *3, and *4 (rs7412 and rs429358) genotypes were determined using LightMix Kit ApoE C112R C158R (TIB MOLBIOL, Germany).

### Ethical statements

The study protocol complied with the Declaration of Helsinki and was approved by the Ethics Committee of the University Hospital Ostrava, Czech Republic. Written informed consent was obtained from each participant.

### Statistical analysis

Statistical analysis was performed using SPSS version 15 (SPSS Inc., Chicago, IL, USA). Continuous clinical variables of the groups with non-normal distribution are presented as the median and range (minimum–maximum) or lower and higher quartiles/Med (LQ–HQ)/ and were compared using the non-parametric Mann–Whitney *U* test. Categorical clinical variables are presented as counts and percentages and were compared by the chi-square test. The genotype distribution of each SNP difference between the study and control groups was analyzed by the chi-square test (or the Fisher’s exact test in the case of lower frequencies). A p value of less than 0.05 was considered significant. Consistency of the observed genotype frequencies with the Hardy–Weinberg distribution was determined by the chi-square test.

Multiple logistic regression was used to evaluate possible effects of other variables on the association observed between the individual SNPs and ISR.

## Results

Basic demographic, clinical, and biochemical characteristics of the cohort are listed in Table 2. The group of patients with ISR and the control group did not differ significantly in the main demographic parameters (age, gender, body mass index) or clinical risk factors (diabetes mellitus). The groups had a similar extent of coronary disease (multi-vessel disease [2VD/3VD], acute coronary syndromes [NSTEMI/STEMI]) and similar lesion characteristics (complex lesion B2/C, length and diameter [±0.5] mm of implanted stents). Furthermore, the group had similar main biochemical parameters (creatinine, TAG, hsCRP). However, total and LDL cholesterol was significantly higher in the control group.

**Table 2:** Clinical characteristics of all patients (including matched controls) and angiographic parameters of coronary artery lesions

	In-stent restenosis	Controls	p
Age (years)	67.0 (59.0–74.0)	67.0 (62.0–71.0)	0.984
Male/Female	101/59	101/59	1.000^b^
Diabetes mellitus (%)	28.75	28.75	1.000^b^
BMI (kg/m2)	28.4 (25.7–31.2)	29.1 (25.9–32.0)	0.368
Creat (umol/L)	97 (86–115)	99 (87–116)	0.517
Chol (mmol/L)	4.27 (3.73–4.94)	4.50 (3.95–5.55)	0.046
TG (mmol/L)	1.62 (1.12–2.18)	1.58 (1.17–2.21)	0.644
LDL (mmol/L)	2.66 (2.27–3.19)	2.96 (2.45–3.67)	0.003
HDL (mmol/L)	1.25 (1.02–1.51)	1.21 (1.00–1.42)	0.354
hs-CRP (mg/L)	1.71 (0.82–3.76)	1.70 (0.85–3.88)	0.978
ACSy (stemi/nstemi) (%)	62.78	72.50	0.056^b^
Multi vessel dissease (%)	57.5	49.38	0.145^b^
B2/C lesions (%)	68.11	68.48	0.940^b^
Stent diameter (mm)	3.0 (2.0–4.5)^a^	3.0 (2.0–4.0)^a^	0.214
Stent lenght (mm)	19 (8–68)^a^	19 (8–86)^a^	0.680

Data are given as median (lower and higher quartiles) or ^a^median (minimim–maximum). Quantitative parameters are given in percents

p, significance of Mann–Whitney *U* test or ^b^chi-square test

In the ISR group, the genotype was determined for all the studied SNPs in 149 patients. Due to lack of DNA, in five patients the genotype was obtained only for rs1803274, rs529038, rs1050450, rs7412, rs429358 and rs7041, in three patients only for rs1803274, rs529038, and rs1050450 and in two patients for rs1803274 and rs529038. In the non ISR group, the genotype was established in all patients for all SNPs with the exception of rs1803274 in 2 patients. Three per cent of the analysis had to be repeated and one per cent to be sequenced.

All SNPs were in Hardy–Weinberg equilibrium. The genotype distributions of the studied polymorphisms and minor allele frequencies (MAF) among subjects with ISR and those without restenosis and MAF data for general Czech population are shown in Table 3. With respect to a small number of minor allele homozygotes of various SNPs, a statistical comparison of homozygotes for the wild-type allele and carriers of the minor allele (heterozygotes and homozygotes) was performed.

**Table 3:** Distribution of polymorphism genotypes in groups with and without ISR, minor alele frequencies (MAF) and logistic regression analysis, separately for each parameter with adjustment for diabetes mellitus

Gene, polymorphism	Distribution of polymorphism genotypes			MAF in general CZ population, ISR and non-ISR groups; statistics			Logistic regression analysis, compared to the wild type allele with adjustment for diabetes mellitus		
	ISR group	Non-ISR group	p	Czech GP	ISR vs. GP	Non-ISR vs GP	OR	95 % CI for OR	p
	No of pts (%)	No of pts (%)			p	p			
*BCHE,* rs1803274									0.016
No of pts (n)	160	158							
GG	102 (63.8 %)	122 (77.2 %)							
GA	50 (31.2 %)	35 (22.2 %)					1.715	1.034 - 2.846	0.037
AA	8 (5 %)	1 (0.6 %)	*0.008* ^a^				*9.703* ^a^	*1.191 - 79.05* ^a^	*0.034* ^a^
GA + AA	58 (36.2 %)	36 (22.8 %)	0.009				1.934	1.181 -3.166	0.009
MAF	0.21	0.12	0.007	0.18	0.280	0.367			
*ROS1,* rs529038									0.280
No of pts (n)	160	160							
GG	75 (46.9 %)	78 (48.8 %)							
GA	75 (46.9 %)	65 (40.6 %)					1.200	0.758 - 1.899	0.436
AA	10 (6.2 %)	17 (10.6 %)	0.269				0.612	0.263 - 1.421	0.253
GA + AA	86 (53.8 %)	82 (51.2 %)	0.823				1.078	0.695 - 1.672	0.737
MAF	0.3	0.31	1	0.3	1	1			
*UPC3,* rs1800849									0.982
No of pts (n)	149	160							
CC	86 (57.8 %)	90 (56.2 %)							
CT	51 (34.2 %)	63 (39.4 %)					0.847	0.527 - 1.361	0.492
TT	12 (8.1 %)	7 (4.8 %)	0.323				1.792	0.670 - 4.793	0.245
CT + TT	63 (42.3 %)	70 (43.8 %)	0.795				0.939	0.597–1.477	0.785
MAF	0,25	0,24	1	0,28	1	0,696			
*GPX1,* rs1050450									0.981
No of pts (n)	157	160							
CC	72 (46 %)	76 (47.5 %)							
CT	74 (47.1 %)	71 (44.5 %)					1.085	0.687 - 1.715	0.726
TT	10 (6.4 %)	13 (8.1 %)	0.788				0.801	0.331 - 1.941	0.623
CT + TT	84 (53.5 %)	84 (52.5 %)	0.811				1.041	0.670 - 1.619	0.857
MAF	0.3	0.3	1	0.23	0.101	0.094			
*ALOX5AP,* rs17216473									1.000
No of pts (n)	149	160							
GG	113 (75.8 %)	120 (75 %)							
GA	36 (24.2 %)	38 (23.8 %)					1.007	0.597 - 1.699	0.980
AA	0	2 (1.2 %)	0.642				-	-	0.999
GA + AA	36 (24.2 %)	40 (25 %)	0.895				0.956	0.569 - 1.605	0.865
MAF	0.12	0.13	1	0.09	0.738	1			
*APOE,* rs7412, rs429358									0.995
No of pts (n)	154	160							
*2/*2	1 (0.6 %)	0					-	-	1.000
2.3	12 (7.8 %)	15 (9.4 %)					0.814	0.363 - 1.826	0.618
2.4	2 (1.3 %)	2 (1.2 %)					1.019	0.141 - 7.369	0.985
*2/*2; *2/*3; *2/*4	15 (9.7 %)	17 (10.6 %)	0.975				0.899	0.427 - 1.896	0.781
3/3 (wild)	106 (68.8 %)	108 (67.5 %)	0.952						
3.4	31 (20.1 %)	32 (20 %)					0.987	0.563 - 1.732	0.964
4.4	2 (1.3 %)	3 (1.9 %)					0.680	0.111 - 4.152	0.676
3/4;4/4	33 (21.4 %)	35 (21.9 %)					0.960	0.556 - 1.658	0.885
MAF *2	0.05	0.05	1	0.08	0.343	0.383			
MAF *4	0.11	0.12	1	0.14	0.831	1			
*GC,* rs7041									0.722
No of pts (n)	154	160							
TT	17 (11 %)	19 (11.9 %)							
TG	83 (53.9 %)	79 (49.4 %)					1.176	0.570 - 2.424	0.661
GG	54 (35.1 %)	62 (38.8 %)	0.741				0.974	0.460 - 2.060	0.945
TG + GG	137 (89 %)	141 (88.1 %)	0.861				1.086	0.542 - 2.178	0.815
MAF	0.38	0.37	1	0.43	0.374	1			
*GC,* rs4588									0.803
No of pts (n)	149	160							
CC	73 (49 %)	82 (51.2 %)							
CA	64 (43 %)	68 (42.5 %)					1.059	0.665 - 1.686	0.810
AA	12 (8.1 %)	10 (6.2 %)	0.821				1.350	0.551 - 3.311	0.512
CA + AA	76 (51.0 %)	78 (48.8 %)	0.733				1.096	0.701–1.713	0.688
MAF	0.29	0.28	1	0.31	1	1			
*VDR,* rs2228570									0.913
No of pts (n)	149	160							
TT	30 (20.1 %)	32 (20 %)							
TC	70 (47 %)	79 (49.4 %)					0.945	0.552 - 1.544	0.760
CC	49 (32.9 %)	49 (30.6 %)	0.9				1.065	0.169 - 3.577	0.747
TC + CC	119 (79.9 %)	128 (80 %)	1				0.991	0.553 - 1.498	0.711
MAF	0.43	0.45	1	0.43	1	1			
*LRP1,* rs1799986									0.913
No of pts (n)	149	160							
CC	109 (73.2 %)	114 (71.2 %)							
CT	37 (24.8 %)	42 (26.2 %)					0.923	0.552 - 1.544	0.760
TT	3 (2.0 %)	4 (2.5 %)	0.941				0.778	0.169 - 3.577	0.747
CT + TT	40 (26.8 %)	46 (28.8 %)	0.8				0.910	0.553 - 1.498	0.711
MAF	0.14	0.15	1	0.16	1	1			
*LDLR,* rs2228671									0.716
No of pts (n)	149	160							
CC	124 (83.2 %)	130 (81.2 %)							
CT	22 (14.8 %)	28 (17.5 %)					0.824	0.447 - 1.516	0.533
TT	3 (2 %)	2 (1.2 %)	0.679				1.579	0.259 - 9.617	0.620
CT + TT	25 (16.9 %)	30 (18.8 %)	0.659				0.874	0.487 - 1.569	0.651
MAF	0.09	0.1	1	0.1	1	1			

*p* = chi-square test

*GP* general population, *CZ* Czech

^a^the confidence interval - for rs1803274 homozygotes AA vs. GG is too wide. Therefore the data were analyzed only in the setting of a dominant model

Evaluation of genotype distributions by the chi-square test (or the Fisher’s exact test in the case of lower frequencies) revealed that only the rs1803274 polymorphism of *BCHE* (c.1699G>A, p.Ala567Thr) was associated with an increased risk of ISR (Table 3). The ISR group had a significantly higher occurrence of heterozygous/homozygous carriers of the A allele (GA + AA) (*p* = 0.009) compared to the control group*.*

Minor allele frequencies (MAF) were calculated in the ISR and the non ISR groups and also in general Czech population. We confirmed a significant difference in rs1803274 MAF between the ISR and non ISR groups (*p* = 0,007). There were no significant differences of MAFs for the other SNPs. No difference was also found between nor ISR neither non ISR group versus general Czech population in any SNP (Table 3).

Multivariate logistic regression analysis with adjustment for the prevalence of diabetes mellitus (the main BMS-ISR clinical risk factor) confirmed that the rs1803274 polymorphism of *BCHE* was significantly associated with the prevalence of ISR (Table 3). The A allele carriers (both heterozygous GA and homozygous AA) were at a 1.934 fold (95 % confidence interval [CI]: 1.181–3.166; *p* = 0.009) increased risk of ISR. Thus, the 1699A allele of the *BCHE* polymorphism represented a risk factor for this condition*.* No association was found between the other SNPs and ISR.

## Discussion

Vascular injury sustained during PCI and bare-metal stent implantation results in a complex inflammatory and reparative process. The acute vascular reaction is characterized by early deposition of platelets and fibrin. Activated platelets attach to circulating leukocytes (neutrophils and monocytes) at the injured surface. Over weeks, acute inflammatory cells are replaced by chronic inflammatory cells (macrophages and giant cells). In addition to this inflammatory response, platelet- and leukocyte-related growth factors drive further VSMC proliferation and migration from the media to the nascent neointima and subsequent extracellular matrix formation.

Two weeks following bare-metal stent implantation, a complete neointimal layer, composed of VSMCs and a proteoglycan-rich extracellular matrix, can be observed above stent struts. Excessive VSMC proliferation and extracellular matrix formation lead to neointimal hyperplasia, which represents the major pathophysiologic mechanism of ISR. Peak of BMS restenosis is observed at 3–6 months and remains relatively stable beyond 1 year [13].

In our study, the ISR and the control groups of patients did not differ significantly in the main demographic, clinical, angiografic and biochemical parameters, with the exception of total and LDL cholesterol levels, that were significantly higher in the control group. However, we do not consider this difference important in the context of the study results, as BCHE is not involved in cholesterol metabolism and no differences were detected in *LDLR* and *LRP1* SNPs between the groups.

Oguri et al. reported that the rs1803274 polymorphism of *BCHE*, rs529038 polymorphism of *ROS1*, and rs1050450 polymorphism of *GPX1* were significantly associated (*p* < 0.05) with ISR and that the rs1800849 polymorphism of *UCP3* was associated with recurrent ISR (*p* = 0.0006) in the Japanese population [3, 4].

Our results show that the rs1803274 (1699G>A, p.Ala567Thr) polymorphism of BCHE was also significantly associated with ISR in the Central European population and that the A allele represents a risk factor for this condition.

We analyzed this association only in the setting of a dominant model, i.e. minor allele homozygotes plus heterozygotes (AA and GA) versus major allele homozygotes (GG), as the confidence interval for rs1803274 homozygotes AA versus GG is too wide, probably due to the low number of AA homozygotes.

A significant difference was also found for rs1803274 minor allele frequencies between the ISR and non ISR groups. However, such a difference was not proved for either the ISR or non ISR groups compared to general Czech population. This may be due to a relatively small sample size.

Butyrylcholinesterase (BCHE) is a secretion enzyme produced by liver cells and released into the bloodstream. In plasma, it contributes to cholinesterase activity, but its endogenous substrate is not known. Cholinesterase synthesis and its activity in plasma are decreased after liver parenchyma damage or in the case of insufficient protein intake in the diet. Previous studies have reported a significant association between serum BCHE activity and metabolic syndrome risk variables, such as high body mass index, plasma concentrations of triglycerides and HDL cholesterol, and blood pressure [14]. BCH E co-participates in the hydrolysis of aspirin and thus could affect its bioavailability and ability to inhibit platelet aggregation [15]. The rs1803274 (the so-called K-variant) SNP could be associated with type 2 diabetes mellitus; on the other hand, results are conflicting on the association between this SNP and early onset of coronary artery disease [16, 17]. The underlying mechanism of the association between the rs1803274 polymorphism of *BCHE* and ISR remains to be elucidated.

Although the rs1050450 polymorphism of the *GPX1* gene has also been associated with an increased risk of ISR in Russian individuals [18] and in the Gender study [19], we found no relationships between the polymorphisms of *ROS1*, *GPX1*, and *UCP3* and ISR in the Central European population.

Arachidonate 5-lipoxygenase-activating protein (ALOX5AP) has an important role in the initial steps of the biosynthesis of leukotrienes, which in turn have a variety of proinflammatory effects [20]. Shah et al. found a significant association between the occurrence of ISR and rs17216473 in a North American population [5]. In contrast to their results, however, we could not confirm the association between rs17216473 and the risk of ISR in a Central European population.

In addition to the above genes, we focused on a group of genes affecting metabolic processes including *APOE, VDR, GC, LRP1* and *LDLR*. The corresponding proteins may be involved not only in atherosclerosis but also in inflammation and may influence proliferation and migration of SMCs, key players in ISR development [6–11].

Apolipoprotein E (APOE) plays an important role not only in the metabolism of cholesterol and triglycerides but also in inhibiting growth factor–induced SMC migration and proliferation and limiting neointimal hyperplasia after arterial injury [6, 7, 21]. APOE deficiency has been associated with increased neointima formation after vessel wall injury [22]. In an apoE-knockout mouse model, the in-stent neointimal area was greater compared with wild-type mice [22]. Although the level of APOE could play a protective role against various forms of vascular disease, including atherosclerosis and injury-induced restenosis, we did not observe a significant correlation between the APOE*2, *3, and *4 polymorphisms and the risk of ISR. Koch et al. made a similar observation, concluding that some *APOE* polymorphisms, i.e., *APOE* –219G>T, 113G>C, 334T>C (APOE*4), and 472C>T (APOE*2) either alone or in combination do not represent genetic markers of the risk of ISR or stent thrombosis in German patients with coronary artery disease [21].

Vitamin D is involved not only in bone metabolism but also in modulating immune responses and cell proliferation. The active form of vitamin D achieves its biological effects by binding to the VDR, which is expressed in most tissues and cells, including cardiac myocytes, VSMCs and endothelial cells [8, 9]. In vivo and in vitro studies have shown that down-regulation of VDR in SMCs of post-interventional arteries could be involved in the uncontrolled growth of SMCs, leading to neointimal hyperplasia and restenosis [10]. We have studied the rs2228570 polymorphism of the *VDR* gene (known as a Fok I variant according to the restriction enzyme used to detect it previously) and the rs7041 and rs4588 polymorphisms of the *GC* gene encoding vitamin D binding protein (DBP) and found that none of these polymorphisms was associated with the risk of ISR in this population*.* The findings regarding the rs2228570 in *VDR* gene correspond to the results of the study by Monraats et al. Although some substitutions (the -1012A>G, −25C>A and 464G>T) of the *VDR* gene increased the risk of clinical restenosis in their study, such an association was not proved for rs2228570 [23].

The members of the low-density lipoprotein receptor family (the LRs) such as low-density lipoprotein receptor (LDLR), low-density lipoprotein receptor-related protein-1 (LRP1), and others play a key role not only in lipoprotein metabolism but also in the catabolism of many membrane-associated proteins [11]. LRs can influence the migration and proliferation of SMCs through urokinase-type plasminogen activator receptor (uPA) and the platelet-derived growth factor (PDGF) receptor. Neither the rs1799986 polymorphism of *LRP1* nor the rs2228671 polymorphism of *LDLR* were significantly associated with the risk of ISR in our study.

### Limitations

Several limitations of our study need to be mentioned. This study could be limited by its observational nature and a relatively small sample size. Nevertheless, we believe that selection bias did not play a major role, because both patient cohorts did not differ with respect to main baseline parameters. As non-invasive MS-CT coronarography was used in control group to confirm the stent patency, it is not possible to avoid completely potential false negatives findings. However, in previous studies, MS-CT coronarography revealed sufficient accuracy for ISR detection [24, 25].

## Conclusion

Our results show that the rs1803274 polymorphism of *BCHE* was significantly associated with ISR in our Central European patients after PCI with bare-metal stent implantation. The A allele of this polymorphism represents a risk factor for this condition. The underlying molecular mechanism of the association between this polymorphism and ISR remains to be elucidated. No association was found with the other studied SNPs, including rs529038 (*ROS1*), rs1050450 (*GPX1*), rs1800849 (*UCP3*), rs17216473 (*ALOX5AP*), rs7412, rs429358 (*APOE*), rs2228570 (*VDR*), rs7041, rs4588 (*GC*), rs1799986 (*LRP1*), and rs2228671 (*LDLR*).

## Availability of supporting data

The data sets supporting the results of this article are available in the LabArchives repository [http://dx.doi.org/10.6070/H4TB14XG].
